# Outcome of Acute Respiratory Failure Secondary to Engraftment in Children After Hematopoietic Stem Cell Transplant

**DOI:** 10.3389/fonc.2020.584269

**Published:** 2020-10-09

**Authors:** Lama Elbahlawan, Ray Morrison, Ying Li, Sujuan Huang, Cheng Cheng, Yvonne Avent, Renee Madden

**Affiliations:** ^1^Division of Critical Care Medicine, St. Jude Children's Research Hospital, Memphis, TN, United States; ^2^Department of Bone Marrow Transplantation and Cellular Therapy, St. Jude Children's Research Hospital, Memphis, TN, United States; ^3^Department of Biostatistics, St. Jude Children's Research Hospital, Memphis, TN, United States

**Keywords:** engraftment syndrome, acute respiratory failure, hematopoietic (stem) cell transplantation (HCT), critically ill, pediatrics

## Abstract

**Introduction:** Respiratory complications due to engraftment syndrome (ES) in the post-hematopoietic stem cell transplant (HSCT) setting can lead to acute respiratory failure (ARF). Outcomes of children developing ARF due to engraftment are unknown.

**Methods:** We conducted a retrospective analysis of 1,527 pediatric HSCT recipients and identified children who developed ARF due to ES over a 17-year period. Thirty patients that developed ARF and required invasive mechanical ventilation (IMV) due to ES were included in this study.

**Results:** The survival rate for our cohort was 80% [alive at intensive care unit (ICU) discharge]. The most common underlying primary disease was hematologic malignancy, and 67% of children underwent allogeneic HSCT. Further, 73% required vasopressor drips and 23% underwent dialysis. Survivors had a shorter median ICU length of stay than did non-survivors (15 vs. 40 days, respectively, *p* = 0.01). Survivors had a significantly lower median cumulative fluid overload % on days 4 and 5 after initiation of IMV than did non-survivors (2.8 vs. 14.0 ml/kg, *p* = 0.038 on day 4, and 1.8 vs. 14.9 ml/kg, *p* = 0.044 on day 5, respectively).

**Conclusion:** Our results suggest that children who develop ARF during engraftment have better ICU survival rates than do those with other etiologies of ARF post-HSCT. Furthermore, fluid overload contributes to mortality in these children; therefore, strategies to prevent and address fluid overload should be considered.

## Introduction

Pulmonary complications after hematopoietic stem cell transplant (HSCT) are relatively common in children and can result in significant morbidity and mortality. Pulmonary injury occurs in 25–55% of pediatric patients receiving HSCT, and transplant-related mortality is as high as 64% (1–3). Such pulmonary complications after HSCT may be attributed to infectious and/or non-infectious lung injury (4). Soon after HSCT, during the period of neutrophil recovery, engraftment syndrome (ES) is one such non-infectious complication that may result in acute respiratory failure (ARF) (5).

Engraftment syndrome has been described in both autologous and allogeneic HSCT (5–18). Although clinical findings of ES may vary, non-specific signs and symptoms commonly associated with ES include non-infectious fever, rash, non-cardiac edema with pulmonary fluid retention, and hypoxia (4). Symptoms usually appear before neutrophil engraftment and have been attributed to a pro-inflammatory condition as a result of the release of cytokines and other inflammatory mediators. This systemic pro-inflammatory state results in the manifestation of clinical findings before the appearance of circulating neutrophils in the peripheral blood. Pulmonary complications during engraftment occur secondary to this cytokine release and capillary leak, which leads to acute lung injury and hypoxia. As a result, respiratory distress with hypoxia can occur and progress to ARF, which requires an escalation of respiratory support to non-invasive mechanical ventilation or invasive mechanical ventilation (IMV).

Pulmonary infiltrates and hypoxia can occur in up to 57% of children with peri-ES post-allogeneic HSCT (19). An earlier report described peri-engraftment respiratory distress and poor survival of adult patients who required IMV (20). Outcomes of pediatric patients receiving HSCT and developing ARF due to engraftment are unknown. The aim of our study is to describe the outcomes of children post-HSCT who experienced ARF and required IMV secondary to engraftment. We also determined the risk factors associated with mortality in this population.

## Patients and Methods

### Study Population

All patients who underwent autologous or allogeneic HSCT and developed ARF requiring IMV between January 2002 and April 2019 were evaluated. The study was approved by the St. Jude Children's Research Hospital Institutional Review Board.

Patients were included if they developed ARF and required IMV during the peri-engraftment period, which included the 7 days preceding and the 7 days after neutrophil engraftment. Neutrophil engraftment was defined as the first day of the 3 consecutive days with an absolute neutrophil count (ANC) ≥0.5 × 10^9^/L. Patients with engraftment syndrome were defined as those with fever, rash, and diffuse non-cardiac pulmonary edema that occurred within 7 days of the neutrophil engraftment date. Patients receiving transplantation after 21 years of age, those with documented pulmonary infection at the time of engraftment, or those with cardiac dysfunction were excluded from this study. Transplantation data collected included the patient's primary diagnosis that required transplant, conditioning regimen, graft source, diagnosis of hepatic veno-occlusive disease, total nucleated cell (TNC) and CD34^+^ cell dose, and administration of colony stimulating factors. Pediatric intensive care unit (PICU) course data abstracted included age, gender, Pediatric Risk of Mortality score, length of IMV, length of PICU stay, use of renal replacement therapy, use of vasopressor support, use of high-frequency oscillatory ventilation (HFOV), use of non-invasive positive pressure ventilation such as bilevel positive airway pressure (BIPAP) or continuous positive airway pressure (CPAP) or high flow oxygen before or after the course of IMV, PICU survival, as well as the 6-month survival. The first week of IMV was examined. Day of initiation of MV was defined as D0 and data were collected within 7 days. Data collected daily included worst PaO_2_/FiO_2_ ratio, worst positive end-expiratory pressure, and worst mean airway pressure (MAP). In addition, daily ANC, C-reactive protein (CRP), albumin, and bilirubin serum levels, as well as daily fluid balance were recorded. Percentage fluid overload was calculated using the following formula: [(Total Fluid Intake in Liters–Total Fluid Output in Liters)/ICU Admission Weight in Kilograms] × 100.

### Statistical Analysis

Descriptive statistics were calculated for demographic and treatment characteristics of patients by intensive care unit (ICU) survival status. Fisher's exact tests and exact Wilcoxon rank-sum tests were used to compare demographic and treatment characteristics between ICU survivors and non-survivors. A two-sided significance level of *p* < 0.05 was used for all statistical tests. The SAS version 9.4 was used for all statistical analyses.

## Results

A total of 1,527 children underwent 2,168 HSCTs during the 17-year period: 878 patients underwent allogeneic transplants and 649 underwent autologous transplants or high-dose chemotherapy followed by stem cell rescue. In these 1,527 patients, 325 episodes of ARF occurred that required IMV in the PICU. Thirty children developed ARF secondary to engraftment and were included in data analysis. ARF secondary to engraftment accounted for 9% of episodes requiring IMV in our PICU, with an incidence of 2% in all children receiving transplants (2.2% in allogeneic vs. 1.5% in autogenic).

### Patient Diagnosis

The patient's primary diagnosis did not have a significant effect on outcomes (Table 1). The most common primary diagnosis in our cohort was hematologic malignancy (50%). Of these 15 patients, seven had acute lymphoblastic leukemia, four had acute myelogenous leukemia, two had lymphoma, and one each had biphenotypic leukemia and juvenile myelomonocytic leukemia. Ten (37%) children underwent transplants for solid tumors (seven had neuroblastoma, two had Ewing sarcoma, and one had medulloblastoma) and five (13%) for non-oncologic diagnosis (two patients had severe aplastic anemia, one patient had Hurler's syndrome, one patient had osteopetrosis, and one patient had severe combined immunodeficiency).

**Table 1:** Clinical characteristics of ARF cohort with ES.

**Characteristic**	**Survivors (*n* = 24)**	**Non-survivors (*n* = 6)**	**Entire cohort (*n* = 30)**	***P*-value**
**GENDER**				1.000
Male	12 (50%)	3 (50%)	15 (50%)	
Female	12 (50%)	3 (50%)	15 (50%)	
**RACE**				0.713
Caucasian	18 (75%)	6 (100%)	24 (80%)	
African-American	4 (17%)		4 (13%)	
Other	2 (8%)		2 (7%)	
**PRIMARY DIAGNOSIS**				0.831
Hematologic malignancy	11 (46%)	4 (67%)	14 (50%)	
Solid tumor	9 (38%)	2 (33%)	11 (37%)	
Other	4 (17%)		4 (13%)	
**BMT TYPE**				1.000
Allogeneic	16 (67%)	4 (67%)	20 (67%)	
Auto	8 (33%)	2 (33%)	10 (33%)	
**G-CSF/GM-CSF**	17 (71%)	2 (33%)	19 (63%)	0.156
**CONDITIONING REGIMEN**				1.000
Myeloablative	16 (67%)	5 (83%)	21 (70%)	
Reduced Intensity	6 (25%)	1 (17%)	7 (23%)	
Non-Myeloablative	2 (8%)		2 (7%)	
TBI	5 (21%)	1 (17%)	6 (20%)	1.000
TNC × 10^8^/kg^a^	2.97 (0.01–79.6)	4.94 (1.5–81)	3.2 (0–81)	0.321
CD34 × 10^6^/kg^a^	7.48 (1.28–103.8)	9.21 (3.18–57.5)	8.4 (1.3–103.8)	0.860
**VOD**	5 (21%)	3 (50%)	8 (27%)	0.300
**Antifungals**	22 (92%)	5 (83%)	27 (90%)	0.501
**Antibiotics**	24 (100%)	6 (100%)	30 (100%)	0.120
**Corticosteroids**	20 (83%)	6 (100%)	26 (87%)	0.557

a *Values are expressed as median with interquartile range*.

*ARF, acute respiratory failure; ES, Engraftment syndrome; BMT, bone marrow transplant; TBI, total body irradiation; TNC, total nucleated cell; VOD, veno-occlusive disease; G-CSF, granulocyte colony stimulating factor; GM-CSF, granulocyte-macrophage colony stimulating factor*.

### Transplant Characteristics

The type of transplant (allogeneic or autologous), TNC dose, or CD34^+^ cell dose did not have a significant effect on survival outcomes of patients with ES in this cohort. The majority (67%) of patients underwent allogeneic HSCT, with nine patients undergoing matched unrelated donor HSCT, eight undergoing haploidentical transplant, and three undergoing a matched related donor transplant. Fifteen (50%) patients received peripheral blood stem cells and nine patients undergoing allogeneic HSCT received peripheral blood stem cell as the graft source. Most of our cohort (70%) received a myeloablative conditioning regimen, and the transplant conditioning regimen or receipt of total body irradiation had no significant effect on the outcomes of ES survivors. The administration of granulocyte colony-stimulating factor/granulocyte-macrophage colony stimulating factor, antimicrobials, or corticosteroids did not differ between survivors and non-survivors.

### ICU Course

Of the 30 children intubated secondary to engraftment, 24 (80%) survived to ICU discharge. The 6-month survival of patients was 62%. Vasopressor support was needed for 73% of patients in our cohort (Table 2). Seven (23%) patients received renal replacement therapy, six were started on CRRT, and one patient was on intermittent hemodialysis that was initiated before the patient developed engraftment-associated ARF.

**Table 2:** ICU course.

	**Survivors (*n* = 24)**	**Non-survivors (*n* = 6)**	***P*-value**
Vasopressor support	16 (66.7%)	6 (100.0%)	0.1550
Dialysis	4 (16.7%)	3 (50.0%)	0.1201
PRISM^a^	13	13.5	0.5512
INO	2 (8.3%)	4 (66.7%)	0.0072
BIPAP/CPAP pre-IMV	1 (4.2%)	2 (33.3%)	0.0936
High flow pre-IMV	13 (54.2%)	1 (16.7%)	0.1686
Duration of ICU stay^a^(d)	15 (7–271)	34 (20–49)	0.03
Duration of IMV^a^(d)	8 (3–30)	33 (5–55)	0.86
**D 0**			
Worse PaO_2_/${\text{FiO}}_{2}^{\text{a}}$	117 (55–363)	55 (50–93)	0.105
Worse ${\text{FiO}}_{2}^{\text{a}}$	80 (40–100)	100 (60–100)	0.177
Worse PEEP^a^	8 (4–16)	8 (5–15)	0.876
Worse MAP^a^	15 (7–28)	18 (14–24)	0.1
**D** **+1**			
Worse PaO_2_/${\text{FiO}}_{2}^{\text{a}}$	164 (72–363)	115 (72–149)	0.105
Worse ${\text{FiO}}_{2}^{\text{a}}$	50 (30–80)	70 (50–100)	0.026
Worse PEEP^a^	9 (4–15)	10 (6–15)	0.758
Worse MAP^a^	18 (9–31)	22 (19–45)	0.012
**D** **+2**			
Worse PaO_2_/${\text{FiO}}_{2}^{\text{a}}$	177 (87–279)	182 (144–230)	0.9
Worse ${\text{FiO}}_{2}^{\text{a}}$	40 (30–100)	50 (40–100)	0.21
Worse PEEP^a^	8 (4–15)	10 (6–15)	0.497
Worse MAP^a^	14 (8–27)	21 (17–45)	0.024
**D** **+6**			
Worse PaO_2_/${\text{FiO}}_{2}^{\text{a}}$	175 (98–444)	98 (53–136)	0.082
Worse ${\text{FiO}}_{2}^{\text{a}}$	40 (27–60)	60 (45–100)	0.054
Worse PEEP^a^	6 (4–12)	7 (6–12)	0.226
Worse MAP^a^	12 (6–22)	24 (20–25)	0.003
**D** **+7**			
Worse PaO_2_/${\text{FiO}}_{2}^{\text{a}}$	175 (103–465)	103 (48–131)	0.039
Worse ${\text{FiO}}_{2}^{\text{a}}$	40 (25–100)	60 (50–100)	0.045
Worse PEEP^a^	5 (5–12)	11 (7–14)	0.081
Worse MAP^a^	11 (8–22)	23 (20–25)	0.1
Cumulative FO % D3^a^	4.1 (−16.6–15.0)	9.5 (2.5–19.9)	0.101
Cumulative FO% D4^a^	2.8 (−17.3–18.5)	14.0 (2.5–30.7)	0.038
Cumulative FO% D5^a^	1.8 (−12.3–19.8)	14.9 (2.5–23.4)	0.044
CRP^a^	18 (0.6–49)	29 (9–34)	0.259
Bilirubin^a^	1.7 (0.2–23.4)	5.5 (1.5–22.4)	0.051
Albumin^a^	3 (2.4–3.6)	3.1 (2.8–3.7)	0.515

a *Values are expressed as median with interquartile range*.

*PRISM, Pediatric Risk of Mortality Score; INO, inhaled nitric oxide; BIPAP, bilevel positive airway pressure; CPAP, continuous positive airway pressure; d; days; IMV, invasive mechanical ventilation; PEEP, positive end expiratory pressure; MAP, mean airway pressure; FO, fluid overload; CRP, C-reactive protein; ICU, intensive care unit*.

Before intubation, 17 (57%) patients required non-invasive MV (three were on BIPAP and 14 on high-flow oxygen). HFOV was used in three patients, of whom two (67%) survived. Survivors had a shorter median length of stay in the ICU than did non-survivors (15 vs. 40 days, *p* = 0.010). In addition, survivors tended to have a shorter course of IMV than did non-survivors (median of 8 days in survivors vs. 33 days in non-survivors, *p* = 0.059).

### Fluid Balance Status

**Figure 1:**
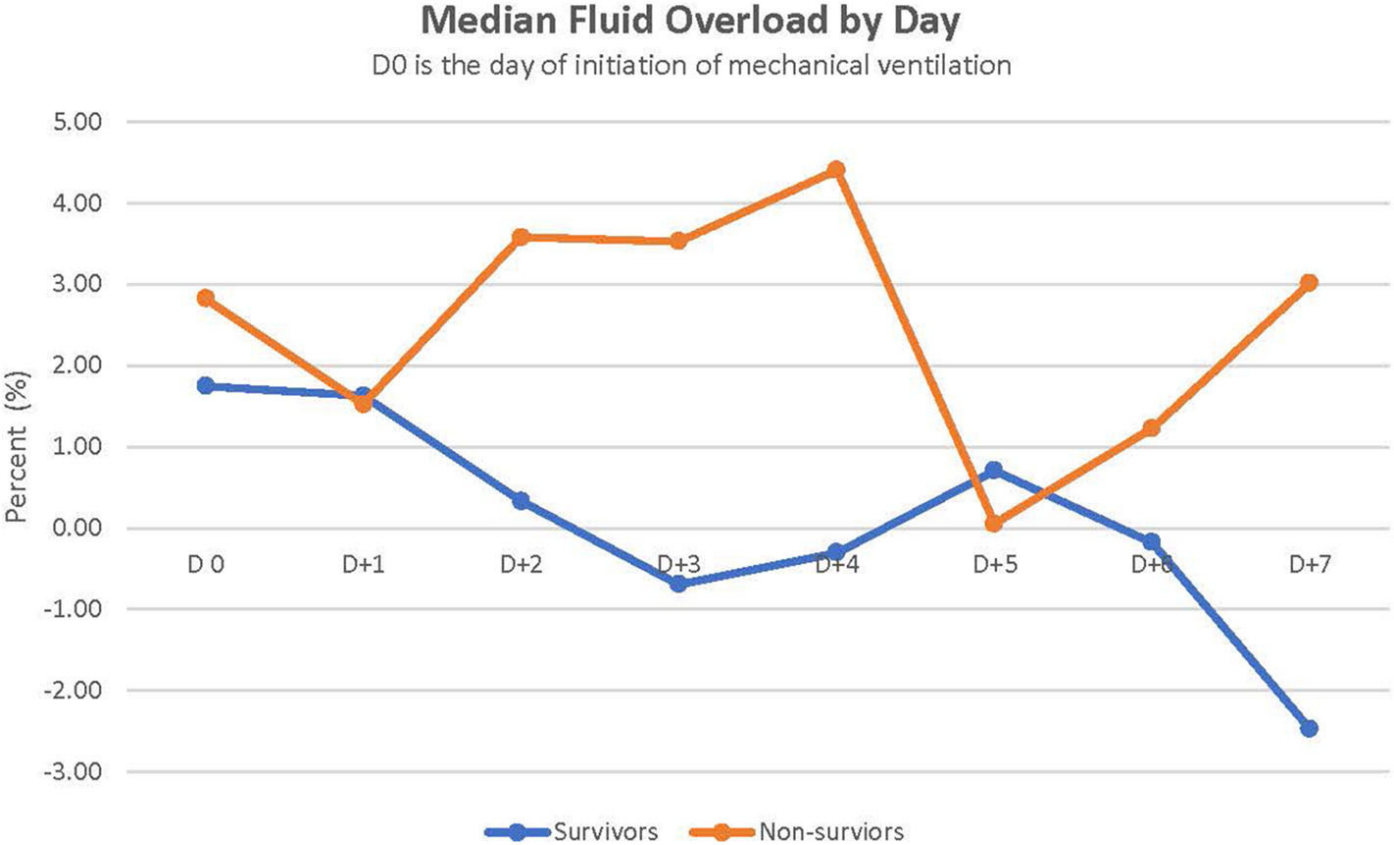
Daily fluid overload % in survivors vs. non-survivors.

Figure 1 depicts the daily fluid status in survivors and non-survivors. Survivors had a lower median cumulative fluid overload % on D4 and D5 of their IMV course than did non-survivors on D4 and D5 (2.8 vs. 14%, *p* = 0.038 on D4; 1.8 vs. 14.9%, *p* = 0.044 on D5) (Table 2). Children with cumulative FO > 20% on D4 as well as D5 had a 7-fold increased risk of dying (RR,7; 95% CI, 2.8–17.3 on D4 and RR,7; 95% CI, 2.7–17.3 on D5).

### Ventilator Settings and Blood Gas Values

On the day of IMV initiation, median MAP in survivors was 15 compared to 18 in non-survivors (*p* = 0.1). During their course of IMV, MAP was significantly higher in non-survivors on D1, D2, and D6 (Table 2). Median PaO_2_/FiO_2_ ratio was 117 in survivors vs. 55 in non-survivors at the time of initiating IMV (*p* = 0.1). The PaO_2_/FiO_2_ ratio was significantly lower in non-survivors than in survivors on D7 (103 vs. 175, *p* = 0.039). Use of inhaled NO was significantly higher in non-survivors than in survivors.

### Lab Values

**Figure 2:**
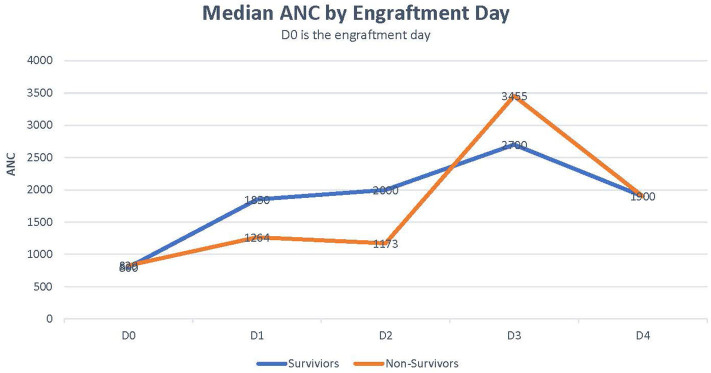
ANC recovery in survivors vs. non-survivors.

Median CRP was 18 in survivors vs. 29 in non-survivors (*p* = 0.259). Median bilirubin level was higher in non-survivors than in survivors (5.5 vs. 1.65, *p* = 0.051; Table 2). Rate of ANC recovery was not significantly different between survivors and non-survivors (Figure 2).

## Discussion

ARF can occur in 25% of pediatric patients receiving HSCT due to heterogeneous etiologies that may be infectious or non-infectious, such as idiopathic pneumonia syndrome, bronchiolitis obliterans, and cryptogenic organizing pneumonia. Peri-engraftment respiratory distress syndrome is a non-infectious pulmonary complication that occurs in the early post-transplant period at the time of neutrophil engraftment and can progress to ARF. We describe herein the largest pediatric cohort that developed ARF with engraftment. The survival rate of 80% in these children is encouraging, considering that the overall survival of children with ARF post-HSCT is reported to be 40–60%. Neutrophil recovery may have contributed to better outcome in our cohort. In a pediatric cohort of intubated HSCT patients, children without neutrophil recovery at the time of intubation who subsequently achieved recovery had ICU survival rate of 61.5% compared to 26% in children whose ANC didn't recover (21). A recent study by Rowan et al. (3) reported a mortality rate of 60.4% in the PICU in a cohort of 222 children with ARF post-HSCT. Impaired oxygenation and higher ventilator pressures were associated with mortality. Similarly, non-survivors in our cohort had higher MAP than did survivors during their IMV course. Previous studies on ARF in the context of engraftment have reported low patient survival rates. Capizzi et al. (20) reported 19 adult patients who developed peri-engraftment respiratory distress syndrome post-autologous HSCT (within 5 days of neutrophil recovery), of whom six patients required IMV and only one patient survived (survival rate 17%). Cahill et al. (22) reported in their cohort that all 14 patients who required MV in the engraftment period died. In our cohort, three patients were put on HFOV, of whom two survived (survival rate 67%). In contrast, Rowan et al. (23) reported a survival rate of only 23.5% in post-allogeneic children who were placed on HFOV. Inhaled NO was used in 25% of patients in our cohort; its use was significantly higher in non-survivors than in survivors. Our observation is similar to that of Rowan et al. (23) but it is not clear whether inhaled nitric oxide (iNO) itself affected survival or this was a mere reflection of the more frequent use of iNO in children with worsening hypoxia when other measures failed, and thus may represent a selection bias. Inhaled nitric oxide is sometimes used for ARF to improve ventilation perfusion mismatch and results in transient improvements in oxygenation, but does not reduce the mortality rates (24). Current evidence does not support the routine use of iNO for ARF, but it can be helpful in children who develop pulmonary hypertension during their course of ARF.

Furthermore, fluid overload was associated with increased mortality in our cohort. Cumulative fluid overload % on day 4 or 5 of the course of IMV was higher in non-survivors than in survivors. This finding is consistent with past reports demonstrating fluid overload as a risk factor for mortality in children with ARF (25, 26). Alobaidi et al. (25) reported that a 1% increase in the percentage of fluid overload resulted in a 6% increase in the odds of mortality in critically ill children. Fluid overload of 10–20% increased the risk of mortality in critically ill children requiring CRRT [odds ratio (OR) 3.83] (26). In addition, more than 20% fluid overload in these critically ill children on CRRT significantly increased mortality (OR = 5) (26). Evidence suggests that fluid overload results in worsening oxygenation and prolonged mechanical ventilation (25). At the time of engraftment, capillary leaks can occur and can significantly contribute to intra-vascular volume depletion. Fluid resuscitation with the ongoing capillary leak subsequently leads to fluid overload. In addition, renal dysfunction can occur during engraftment in up to 10% of patients (19). Diuretics are usually administered intermittently or as a continuous drip to improve diuresis and fluid removal. However, even with aggressive use of diuretics, fluid overload may continue. Continuous renal replacement therapy (CRRT) may be needed to reduce fluid overload (27). Indeed, 7/30 (23%) children of our cohort required CRRT, of whom four survived. CRRT can improve oxygenation in children post-HSCT with ARF and help reduce fluid overload (28). In a cohort of 161 children treated with CRRT, critically ill children with onco-hematologic disease had the highest mortality (78% compared to 36% overall mortality in the entire cohort) (26). A survival of 57% in our cohort is encouraging.

Factors associated with the risk of ES in allogeneic or autologous transplant recipients have included female gender, high graft TNC count or CD34 cell count, underlying malignancy, and conditioning regimen (5, 10, 11, 15, 16). In our small cohort, no significant difference between survivors and non-survivors was seen with regard to these factors or with the administration of growth factors and antimicrobials. Additionally, autologous or allogeneic HSCT did not appear to affect PICU outcomes in this limited patient cohort (20 vs. 25% non-survivors, respectively).

The majority of our cohort (87%) received steroids; therefore, it is difficult to determine whether steroids affected the outcomes. Steroids are thought to reduce the inflammatory response and the resultant capillary leak and often used in the setting of engraftment syndrome. A short course of steroids for 3–5 days was effective in children post-autologous transplant and did not increase the risk of infection (29).

The limitations of our study include its retrospective design, small population, and absence of a control group. However, our study describes the largest cohort of 30 children post-HSCT who developed ARF secondary to engraftment over a span of 17 years. Prospective multi-center studies analyzing specific etiologies of ARF after HSCT are warranted, given the heterogeneity in courses, outcomes, and factors influencing survival.

## Conclusion

Our findings show better survival rates for children with ARF secondary to engraftment post-HSCT. Fluid status is particularly important and requires attention during this phase. Our findings suggest that fluid overload can increase the risk of mortality in this population, and measures to prevent fluid overload may help improve outcomes.

## Data Availability Statement

The raw data supporting the conclusions of this article will be made available by the authors, without undue reservation.

## Ethics Statement

The studies involving human participants were reviewed and approved by IRB at St Jude Children's Research Hospital. Written informed consent from the participants' legal guardian/next of kin was not required to participate in this study in accordance with the national legislation and the institutional requirements.

## Author Contributions

LE and RMa: conceptualization. LE, SH, CC, YA, and RMa: data curation, project administration, and validation. LE, RMo, SH, CC, and RMa: formal analysis, visualization, and writing—original draft. LE, RMo, YL, SH, CC, YA, and RMa: investigation, methodology, and writing—review and editing. All authors have reviewed and approved the final manuscript as submitted and agreed to be accountable for all aspects of the work. All authors contributed to the article and approved the submitted version.

## Conflict of Interest

The authors declare that the research was conducted in the absence of any commercial or financial relationships that could be construed as a potential conflict of interest.
